# Association between rectal colonization with Highly Resistant Gram-negative Rods (HR-GNRs) and subsequent infection with HR-GNRs in clinical patients: A one year historical cohort study

**DOI:** 10.1371/journal.pone.0211016

**Published:** 2019-01-25

**Authors:** Dennis Souverein, Sjoerd M. Euser, Bjorn L. Herpers, Jan Kluytmans, John W. A. Rossen, Jeroen W. Den Boer

**Affiliations:** 1 Department of Epidemiology and Infection Prevention, Regional Public Health Laboratory Kennemerland, Haarlem, the Netherlands; 2 Laboratory for Microbiology and Infection Control, Amphia Hospital, Breda, The Netherlands; 3 University Medical Center, Utrecht, the Netherlands; 4 Department of Medical Microbiology, University of Groningen, University Medical Center Groningen, Groningen, the Netherlands; Universitatsspital Basel, SWITZERLAND

## Abstract

**Objective:**

Rectal colonization with Highly Resistant Gram-negative Rods (HR-GNRs) probably precedes infection. We aimed to assess the association between rectal HR-GNR colonization and subsequent HR-GNR infection in clinical patients during a follow-up period of one year in a historical cohort study design.

**Methods:**

Rectal HR-GNR colonization was assessed by culturing. Subsequent development of infection was determined by assessing all clinical microbiological culture results extracted from the laboratory information system including clinical data regarding HR-GNR infections. A multivariable logistic regression model was constructed with HR-GNR rectal colonization as independent variable and HR-GNR infection as dependent variable. Gender, age, antibiotic use, historic clinical admission and previous (HR-GNR) infections were included as possible confounders.

**Results:**

1133 patients were included of whom 68 patients (6.1%) were colonized with a HR-GNR. In total 22 patients with HR-GNR infections were detected. Urinary tract infections were most common (n = 14, 63.6%), followed by bloodstream infections (n = 5, 22.7%) and other infections (n = 8, 36.4%). Eight out of 68 HR-GNR colonized patients (11.8%) developed a subsequent HR-GNR infection compared to 14 out of 1065 HR-GNR negative patients (1.3%), resulting in an odds ratio (95% CI) of 7.1 (2.8–18.1) in the multivariable logistic regression analyses.

**Conclusions:**

Rectal colonization with a HR-GNR was a significant risk factor for a subsequent HR-GNR infection.

This implies that historical colonization culture results should be considered in the choice of empirical antibiotic therapy to include coverage of the cultured HR-GNR, at least in critically ill patients.

## Introduction

Infections are frequently seen by general practitioners (GPs) and medical specialists in the Netherlands [1]. In general, a rapid diagnosis and effective empirical antibiotic therapy are important to prevent complications. Examples are, pyelonephritis or bloodstream infection (BSI) in patients with a urinary tract infection (UTI) [2]. However, rising resistance rates hamper effective antibiotic treatment increasing the risk of treatment failure and complications [3]. Clinical infections with Highly Resistant Gram-negative Rods (HR-GNR) are associated with an increased risk of morbidity, mortality, and healthcare costs compared to susceptible micro-organisms [4–8].

It has been hypothesized that many infections, such as UTIs and BSIs emerge from an intestinal reservoir [9, 10]. We wanted to determine if rectally HR-GNR colonized patients are at increased risk of developing a HR-GNR infection. These insights could be helpful in timely application of the most appropriate antibiotic treatment. When rectal HR-GNR colonisation is associated with an increased risk of a subsequent HR-GNR infection, identification of colonized patients and prevention of colonization becomes more important, because of the implications that it will have for patients. In order to study this association the present study was designed.

Between 2013 and 2015, an annual HR-GNR rectal colonization prevalence measurement was performed in three hospitals in the Dutch region Kennemerland as part of each hospitals infection control program [11]. The present study was designed as a historical cohort study aiming to assess the association between rectal HR-GNR colonization and subsequent HR-GNR infection over a follow-up period of one year in the clinical patient population.

## Methods

### Ethics statement

According to the Dutch regulation for research with human subjects, neither medical or ethical approval was required to conduct the study since the data were collected as part of routine diagnostics and each hospitals infection control program. The data were analysed under code.

### Study design, setting and participants

The present study was designed as a historical cohort study. Eligible patients were all patients who participated in an annually (between 2013 and 2015) performed cross-sectional (point)prevalence measurement within three regional hospitals in the Dutch region Kennemerland. Within this prevalence measurement the rectal HR-GNR colonization status was assessed by analysing a rectal swab for the presence of HR-GNRs. Rectal swabs were obtained from hospitalized patients (independent of the hospitalization time) at all participating wards: internal medicine, cardiology, neurology, surgery, urology, pulmonology, intensive care unit (ICU), paediatrics, geriatrics, orthopaedics and gynaecology as part of each hospitals infection control program. Outpatients as well as patients on day care were excluded. Patients were considered at risk of HR-GNR infection starting on the day of the HR-GNR prevalence measurement until one year after their enrolment.

### Definition of HR-GNR

The group of Highly Resistant Gram-negative Rods (HR-GNRs) is defined as (1) Enterobacteriaceae that are Extended Spectrum Beta-Lactamase (ESBL) and/or carbapenemase positive (CPE) and/or resistant towards Fluoroquinolones and Aminoglycosides (Q&A), (2) *Acinetobacter* spp. that are carbapenemase positive and/or resistant to Q&A, (3) *Stenotrophomonas maltophilia* resistant to co-trimoxazole and (4) multi-resistant *Pseudomonas aeruginosa*, defined as resistant to at least three of the following antibiotics or antibiotic groups: piperacillin, ceftazidime, fluoroquinolones, aminoglycosides and/or carbapenemase positive.

### Baseline detection of HR-GNRs in rectal swabs

Rectal swabs (Copan eSwab including 1 mL of modified liquid Amies) were analysed for the presence of HR-GNRs at the RPHLK by direct culturing on both an ESBL screening agar (ChromID ESBL-ID, bioMerieux, enriched with a mixture of antibiotics, including cefpodoxime) and a CLED GM20 agar (with 20 mg/L gentamicin, Oxoid) and incubated overnight at 37°C. Gram-negative rods growing on these two agars were identified using MALDI-TOF (Bruker Daltonics, Germany). Antibiotic susceptibility testing was performed using the automated system VITEK2 (bioMérieux, France) using CLSI breakpoints. All isolates suspected for the production of ESBL, defined as a VITEK 2 AES alert and/or elevated MIC (> 1 mg/L) for cefotaxime and/or ceftazidime were confirmed using the combination disk method (ceftazidime and cefotaxime or cefepime with and without clavulanic acid) [12]. Bacteria with a VITEK 2 AES alert and/or elevated MIC for meropenem (> 0.25 mg/L) were suspected for carbapenemase production and confirmed using the modified Hodge test [12]. All positive isolates were stored at -80°C. Molecular characterization of baseline HR-GNR isolates was performed by Whole Genome Sequencing (WGS) based on earlier described methods [13–16].

### Exposure definition

Rectal HR-GNR colonization status was used as exposure variable. Patients were classified as rectal HR-GNR colonized when culturing HR-GNR positive during one of the prevalence measurements. For patients, who tested HR-GNR positive more than once, only the first result was included. For patients who tested negative, the first HR-GNR negative rectal culture result was included.

### Outcome definition

In order to determine subsequent (HR-GNR) infections, for every patient, all microbiological culture results over a follow-up period of one year, after the baseline HR-GNR measurement were extracted from the Laboratory Information System (LIS) of the Regional Public Health Laboratory Kennemerland (RPHLK) including cultures from general practitioners, nursing homes and hospitals in the region Kennemerland. These clinical samples were collected as part of routine clinical management (according to the treating physician) and analysed with standard microbiological culture methods. Based on these results, patients were classified as not infected when no culture result was found, also when a positive culture was found from a non-sterile body site (such as the rectum, throat or nose) since these represents colonization instead of infection. All samples that were marked as infected are described in Table A in S1 File. An UTI was defined as a positive (non-catheter) urine culture, growing more than 10^3^ colony forming units per mL (CFUs), including the growth of uropathogens (such as *E*. *coli* or *K*. *pneumoniae*), according to international guidelines [17]. For all cultures with growth of HR-GNRs the clinical microbiologist determined which cases represented actual infection, by reviewing the clinical data on symptoms, diagnosis and treatment in the electronic health record. After reviewing all microbiological culture results and clinical information, patients were classified as uninfected or (HR-GNR) infected. Patients could have more than one infection classified into UTI, BSI and/or other infections. For all patients with infections, the site of infection and HR-GNR type was registered.

### Potential confounders and effect modifiers

The following data were obtained at baseline from the hospital (HIS) and laboratory information system (LIS) in order to correct for possible confounders or stratify for possible effect modifiers: gender, age (in years), ICU admission (yes/no), antibiotic use up to 6-months before baseline (yes/no), antibiotic use at baseline (yes/no), earlier clinical admissions up to one year before baseline (yes/no), earlier HR-GNR infections up to two years before baseline and time from admission to sampling at baseline (in days). Historic microbiological culture results were used to determine earlier (HR-GNR) infection(s) based on the same definitions of our outcome. Based on earlier research these variables could possibly bias our studied association between HR-GNR colonization and subsequent HR-GNR infection and were therefore incorporated in the multivariable logistic regression model as confounder and possible effect modifier.

### Descriptive and statistical analyses

Baseline characteristics, rectal HR-GNR colonization prevalence and HR-GNR infection(s) during follow-up were first descriptively analysed and presented as numbers and percentages. In order to study the association between rectal HR-GNR colonization and subsequent risk of HR-GNR infection over a follow-up period of one year, a multivariable logistic regression model was constructed. After the crude logistic regression analysis, all possible confounders were first analysed univariate and then included one-by-one when the regression coefficient changed with more than 10% after correction (strongest first) as suggested by Twisk [18]. In order to minimize residual confounding for possible continuous confounders (age and time from admission to sampling) these were first checked for a linear association with the outcome, and when not linearly associated, analysed as quartiles. After correction for possible confounders, the model was checked for possible effect modifiers by analysing the model with interaction terms and stratified when significant. The unadjusted and adjusted association was presented as an odds ratio, including a 95% confidence interval and p-value. All analyses were performed using IBM SPSS Statistics version 25.0. Results were interpreted as statistically significant when the p-value was < 0.05.

## Results

### Participants

Between 2013 and 2015, 1133 patients were included as they were screened in one of the three HR-GNR prevalence measurements. Out of these 1133 patients, 68 patients (6.1%) were identified as rectally colonized with a HR-GNR. As shown in Table 1, from the majority of colonized patients (n = 57, 83.8%) an ESBL producing micro-organism was isolated, followed by Q&A (n = 13, 19.1%) and CPE (n = 1, 1.5%). Three patients (1.0%) were colonized with more than one type of HR-GNR. In total, 76 HR-GNR micro-organisms from 68 patients were further characterized. A description of these isolates is given in Table B in S1 File. *E*. *coli* (n = 57, 75.0%) was found most frequently followed by *K*. *pneumoniae* (n = 11, 14.5%), *E*. *cloacae complex* (n = 7, 9.2%) and *M*. *morganii* (n = 1, 1.3%). The baseline characteristics of the cohort, are reported in Table 1.

**Table 1:** Baseline characteristics of the cohort.

Patient characteristics	Total (n = 1133)	HR-GNR colonized (n = 68)	HR-GNR not colonized (n = 1065)
Number of HR-GNR colonized patients	68 (6.1)	68 (100)	-
ESBL positive	57 (5.0)	57 (83.8)	-
Q&A positive	13 (1.1)	13 (19.1)	-
CPE positive	1 (0.1)	1 (1.5)	-
Other positive	0 (0)	0 (0)	-
Sex (male)	553 (48.8)	36 (52.9)	517 (48.5)
ICU admission	61 (5.4)	6 (8.8)	55 (5.2)
Used antibiotics 6 months before baseline	223 (19.7)	21 (30.9)	202 (19.0)
Used antibiotics at baseline	496 (43.8)	37 (54.4)	459 (43.1)
Admitted before baseline (up to 1 year)	524 (46.2)	43 (63.2)	481 (45.2)
Earlier infection (up to two years before baseline)	293 (25.9)	35 (51.5)	258 (24.2)
Mean age in years (SD)	63.3 (22.9)	68.2 (16.3)	63.0 (23.2)
Median time from start admission to sampling in days (range) at baseline	3.0 (0–74)	5.5 (0–48)	3 (0–74)

Data are presented as numbers (%) unless indicated otherwise.

HR-GNR: Highly Resistant Gram-negative Rod; ESBL: extended spectrum beta lactamase; Q&A: Enterobacteriaceae resistant to fluoroquinolones and aminoglycosides; CPE: carbapenemase producing Enterobacteriaceae.

### Descriptive analysis

Out of 1133 patients, 201 patients were identified with one or more infections (239 infections) over the total follow-up period of one year, resulting in a cumulative incidence of 17.7% (95% CI: 15.6–20.1) (Table 2). Within this group of patients, UTI was most common (n = 145, 72.1%), followed by BSI (n = 47, 23.4%) and other infections (n = 47, 23.4%). The group of other infections included cultures with micro-organisms isolated from: pus, body tissue material and normally sterile body fluid such as cerebrospinal fluid and pleural fluid. Twenty-nine patients (14.4%) were identified with more than one infection (counted as different episodes), of whom 22 patients (75.8%) had both an UTI and a BSI.

**Table 2:** Descriptive subsequent (HR-GNR) infection(s) of the cohort.

	Total (n = 1133)	HR-GNR colonized (n = 68)	HR-GNR not colonized (n = 1065)
Subsequent infection(s)	201 (17.7)	17 (25.0)	184 (17.3)
UTI	145 (12.8)	15 (22.1)	130 (12.2)
BSI	47 (4.1)	2 (2.9)	45 (4.2)
Other	47 (4.1)	4 (5.9)	43 (4.0)
More than one infection	29 (2.6)	3 (4.4)	26 (2.4)
Both UTI and BSI	22 (1.9)	2 (2.9)	20 (1.9)
Subsequent (HR-GNR) infection(s)	22 (1.9)	8 (11.8)	14 (1.3)
UTI	14 (1.2)	5 (7.4)	9 (0.8)
Uncomplicated UTI	6 (0.5)	3 (4.4)	3 (0.3)
Complicated UTI	8 (0.7)	2 (2.9)	6 (0.6)
BSI	5 (0.4)	1 (1.5)	4 (0.4)
Urosepsis	3 (0.3)	0 (0.0)	3 (0.3)
Other BSI	2 (0.2)	1 (1.5)	1 (0.1)
Other	8 (0.7)	3 (4.4)	5 (0.5)
Wound infection	6 (0.5)	2 (2.9)	4 (0.4)
Other infections	2 (0.2)	1 (1.5)	1 (0.1)
More than one HR-GNR infection	5 (0.4)	1 (1.5)	4 (0.4)

Data are presented as number of patients (%)

UTI: urinary tract infection; BSI: bloodstream infection; Other: other infection than UTI or BSI.

Out of 201 patients with an infection, 22 patients (10.9%) were identified with one or more infections (27 infections) with a HR-GNR micro-organism. These infections were most frequently identified within the hospital (n = 21, 77.8%), followed by the general practitioner (n = 3, 11.1) and the nursing home (n = 3, 11.1). UTI was most common (n = 14, 63.6%), followed by BSI (n = 5, 22.7%) and patients with another infection than UTI and/or BSI (n = 8, 36.4%). Five patients (22.7%) were identified with more than one HR-GNR infection within the follow-up period, of whom three with urosepsis. Characteristics of HR-GNR colonized patients (with or without HR-GNR infection) are reported in Table C in S1 File. Number and type of (HR-GNR) infection(s) between HR-GNR colonized and not colonized patients are reported in Table D in S1 File.

### Crude analysis of infection after colonization

Seventeen out of 68 HR-GNR colonized patients (25.0%) developed a subsequent infection (independent of the resistance profile) compared to 184 out of 1065 HR-GNR negative patients (17.3%) resulting in an odds ratio (95%CI) of 1.6 (0.9–2.8). Comparing subsequent HR-GNR infections between HR-GNR colonized and negative patients showed that eight out of 68 HR-GNR colonized patients (11.8%) developed a subsequent HR-GNR infection, compared to 14 out of 1065 HR-GNR negative patients (1.3%). Crude logistic regression analysis resulted in an odds ratio (95% CI) of 10.0 (4.0–24.8) showing that rectal HR-GNR colonization is a significant risk factor for a subsequent infection with a HR-GNR micro-organism. Table 3 shows the association between HR-GNR colonization for UTI, BSI and other infections.

**Table 3:** Association between HR-GNR colonization and subsequent HR-GNR infection.

Outcome	HR-GNR colonized (n = 68)	HR-GNR not colonized (n = 1065)	Unadjusted Odds ratio (95% CI)	Unadjusted p-value	Adjusted Odds ratio (95% CI) ^1^	Adjusted p-value ^1^
Subsequent HR-GNR infection	8 (11.8)	14 (1.3)	10.01 (4.04–24.79)	<0.001	7.07 (2.76–18.08)	<0.001
Subsequent HR-GNR infection (urine)	5 (7.4)	9 (0.8)	9.31 (3.03–28.61)	<0.001	6.00 (1.88–19.13)	0.002
Subsequent HR-GNR infection (blood)	1 (1.5)	4 (0.4)	3.96 (0.44–35.92)	0.221	2.72 (0.29–26.00)	0.385
Subsequent HR-GNR infection (other)	3 (4.4)	5 (0.5)	9.79 (2.29–41.84)	0.002	6.92 (1.54–31.17)	0.012
Subsequent infection (all bacteria and infection sites)	17 (25.0)	184 (17.3)	1.60 (0.90–2.83)	0.109	1.10 (0.60–2.01)	0.754

HR-GNR: Highly Resistant Gram-negative Rod

^1^: corrected for an earlier infection (up to two years before baseline)

### Multivariable analysis of HR-GNR infection after colonization

Within the multivariable logistic regression model, only a historic infection (up to two-years before the HR-GNR prevalence measurement) changed the regression coefficient with more than 10% and was therefore considered as a relevant confounder. No other confounders and significant effect-modifiers were identified. The adjusted model showed an odds ratio (95% CI) of 7.1 (2.8–18.1) (Table 3).

## Discussion

In the present study, we aimed to assess the association between rectal HR-GNR colonization and subsequent HR-GNR infection over a follow-up period of one year in the clinical patient population. We analysed 1,133 patients of whom 68 patients (6.1%) were rectally colonized with a HR-GNR micro-organism. Eight out of 68 HR-GNR colonized patients (11.8%) developed a subsequent HR-GNR infection compared to 14 out of 1065 HR-GNR negative patients (1.3%), resulting in an odds ratio (95% CI) of 7.1 (2.8–18.1) in the multivariable logistic regression analyses.

Earlier studies analysed similar associations between colonization and subsequent infection with several micro-organisms other than HR-GNRs. Many studies showed that nasal MRSA colonization was an independent risk factor for a subsequent MRSA infection [19, 20]. Another study showed that colonization with *K*. *pneumoniae* increased the risk (odds ratio 4.1) for infection with *K*. *pneumoniae* (including UTI, BSI and pneumonia) [10]. This result was confirmed within ICU patients with an odds ratio of 6.9 [21]. In both studies all *K*. *pneumoniae* isolates were included, also those that were completely susceptible to all empirical antibiotics. For *Pseudomonas aeruginosa* colonization and subsequent infection an incidence rate ratio of 6.74 was found by Harris et al. [22]. Platteel et al. found that 14% of intestinal ESBL carriers compared to 0.4% of non-carriers developed an infection during admission with ESBL-producing bacteria [23]. However, no type of infection was reported and no multivariable analysis on this association was performed. One other study found little evidence that ESBL screening at admission could be helpful in the clinical decision for treating BSI [24]. In our study, we included HR-GNRs, mainly consisting of Enterobacteriaceae that were ESBL and/or Q&A positive. We found a similar association compared to the previously mentioned studies and confirm the conclusion that colonization is an important step before infection.

From this and other studies, several lessons can be learned. First, infections are frequently endogenous with the patient’s own intestinal micro-flora as important reservoir. Second, it is important to prevent nosocomial colonization with HR-GNRs as these pose an increased risk to subsequent infections. Third, when HR-GNR colonized patients present themselves with clinical signs of an infection, such as an UTI, physicians should realize that these patients are at increased risk of an infection with a HR-GNR micro-organism. Choosing an appropriate antibiotic therapy based on the colonizing micro-organism, could possibly reduce treatment failures and complications in this patient group. Fourth, decolonization of colonized patients has been suggested by several studies, but seems difficult as most HR-GNR micro-organisms (mostly Enterobacteriaceae) are normal inhabitants of the intestinal tract [25, 26].

Our study has several strengths. In order to determine subsequent infections, we used the extensive LIS of the central microbiology laboratory in the region Kennemerland including clinical data for reviewing subsequent HR-GNR infection. We expected that when a culture was performed for our study population during the follow-up period, these results would be present in the LIS, as the RPHLK is a microbiology diagnostic and expertise laboratory that performs all infectious disease diagnostics for general practitioners, nursing homes and hospitals in the Dutch region Kennemerland. Using this approach, we were also able to determine historical (HR-GNR) infections and use this information in our multivariable logistic regression model. For the total group of infections we also made a clear distinction in the patient materials to define an infection. For instance, patients with positive culture results from urine derived from a catheter were excluded as catheters are frequently colonized without infection [27]. Furthermore, our population is comprised of patients from all hospital wards increasing the generalizability compared to most other studies that only included specific wards such as ICUs. Finally, no comparable studies were found that studied the risk of subsequent HR-GNR infection after colonization.

Our study has also some important limitations. We only incorporated clinical data in the outcome definition of subsequent HR-GNR infection. For the large group of subsequent non-HR-GNR infection(s) we used laboratory data only. when no positive culture was found patients were classified as not infected in the LIS of the RPHLK. For this group uncertainty existed in the presence or absence of infection. Also, it was possible, that for some patients who developed an infection no culture results were known, leading to misclassification as these patients were classified as not infected. However, the baseline HR-GNR rectal colonization measurement (the independent ‘exposure’ variable) was performed anonymously and no result was reported back to patient and physician. We therefore expect that this type of misclassification, when present was non-differential. Also, physicians may perform clinical culturing less strictly when patients are known with earlier infections with a HR-GNR micro-organism leading to more clinical culturing in this group. These data were incorporated and controlled for in our multivariable model as we incorporated historic microbiological results. Given the low prevalence of HR-GNR colonization and the low number of HR-GNR infections our possibilities to build a robust association model was limited. Since we found similar associations as in other colonization infection studies we have confidence that this reported association is correct. Furthermore, no WGS was performed on HR-GNR infection isolates as these were not available for WGS, limiting the genetic comparison with HR-GNR colonization isolates, on which WGS was conducted. So, there is some uncertainty that the infection was caused by the colonizing isolate. Although WGS might had given more insight in the pathogenic mechanisms involved, it would not have influenced the studied association as HR-GNR colonized patients would still have a higher risk of a subsequent infection with a HR-GNR micro-organism. Also, the usefulness of a genetic comparison between colonizing and infection isolates could be questioned as studies showed that colonization with certain sequence types could vary over time biasing the comparison when no genetic relation was found [28].

Our results indicate that routine screening could probably help in individual patient care. Combining our results with previously identified risk factors for a HR-GNR infection could possibly help to develop a region wide clinical decision rule for appropriate antibiotic prescription. Earlier studies tried to develop such a prediction rule, to identify patients with BSI caused by third-generation cephalosporin (3GC)–resistant Enterobacteriaceae (3GC-R EB) [29, 30]. Therapy based on earlier 3GC-R EB positive cultures alone (infection or colonization) will stimulate unnecessary carbapenem use as these models show limited predictive values, which is undesirable [29]. However, when carbapenem therapy is indicated based on colonizing culture results, at least in critically ill patients, antimicrobial choice should always be reassessed as soon as infection culture results become available in order to limit resistance development and adverse effects. Prediction rules should also include infections other than BSI as outcome since a recent study showed that more than 50% of *E*. *coli* BSI had a focus in the urogenital tract [31]. Preventing BSI in this population by an adequate and effective therapy opens the possibility for better patient outcomes. Within hospitals, patients at risk for infections could be screened upon admission, as is already common practice in various hospitals [32–34]. A possible application for targeted screening and therapy is with a TRUS biopsy procedure where antibiotics are prescribed prophylactically in order to prevent infection [35]. Physicians can only take full advantage of these results when microbiology data are regionally available for all treating physicians such as general practitioners, nursing home physicians, and hospital physicians. Recently published results showed that patients are frequently transferred within regions and consulted by several health care providers [36]. Therefore, sharing microbiology data has not only benefits for individual patients but also on a population level, since transmission of antimicrobial resistance is not restricted to one health care provider.

In conclusion, rectal colonization with a HR-GNR was a significant risk factor for a subsequent HR-GNR infection within this clinical patient population. This implies that historical colonization culture results should be considered in the choice of empirical antibiotic therapy to include coverage of the cultured HR-GNR, at least in critically ill patients.

## Supporting information

S1 File Table A, Table B, Table C and Table D.
(DOCX)
