# Supplementary material for: Association between rectal colonization with Highly Resistant Gram-negative Rods (HR-GNRs) and subsequent infection with HR-GNRs in clinical patients: A one year historical cohort study
Source: PLoS One. 2019 Jan 25;14(1):e0211016. doi: 10.1371/journal.pone.0211016 (PMC6347189; doi:10.1371/journal.pone.0211016)
Supplement: S1 File — Table A, Table B, Table C and Table D. (DOCX) [file pone.0211016.s001.docx]

**Supporting information**

**Table A** Definition, handling and use of patient material used for culturing

| Culture site | Condition | Marked as |
| --- | --- | --- |
| Blood | All micro-organisms and growth quantity ^1^ | Blood infection |
| Catheter tip | All micro-organisms > 15 CFU | Blood infection |
| Urine (non-catheter) | Pathogenic bacteria (such as *E. coli* or *K. pneumoniae*) > 10^3^ CFU | Urine infection |
| Abscess | All micro-organisms and growth quantity | Other Infection |
| Ascites fluid | All micro-organisms and growth quantity ^1^ | Other Infection |
| Biopsy | All micro-organisms and growth quantity | Other Infection |
| Lymph node | All micro-organisms and growth quantity | Other Infection |
| Cerebrospinal fluid | All micro-organisms and growth quantity ^1^ | Other Infection |
| Pleural fluid | All micro-organisms and growth quantity ^1^ | Other Infection |
| Pus | All micro-organisms and growth quantity ^1^ | Other Infection |
| Urine (catheter) | Pathogenic bacteria (such as *E. coli* or *K. pneumoniae*) > 10^3^ CFU | No infection/colonization/possible infection |
| Bronchoalveolar lavage | All micro-organisms and growth quantity | No infection/colonization/possible infection |
| Eye | All micro-organisms and growth quantity | No infection/colonization/possible infection |
| Ear | All micro-organisms and growth quantity | No infection/colonization/possible infection |
| Genitals | All micro-organisms and growth quantity | No infection/colonization/possible infection |
| Sputum | All micro-organisms and growth quantity | No infection/colonization/possible infection |
| Ulcer | All micro-organisms and growth quantity | No infection/colonization/possible infection |
| Rectal swab/feces | All micro-organisms and growth quantity | No infection/colonization/possible infection |
| Skin | All micro-organisms and growth quantity | No infection/colonization/possible infection |
| Wound | All micro-organisms and growth quantity | No infection/colonization/possible infection |
| Cervix | All micro-organisms and growth quantity | No infection/colonization/possible infection |
| Throat | All micro-organisms and growth quantity | No infection/colonization/possible infection |
| Mouth | All micro-organisms and growth quantity | No infection/colonization/possible infection |
| Nasopharynx | All micro-organisms and growth quantity | No infection/colonization/possible infection |
| Nose | All micro-organisms and growth quantity | No infection/colonization/possible infection |

1: Clinically non-relevant micro-organisms originating from contamination of the specimen (for example skin bacteria) were excluded, as assessed by the clinical microbiologist

**Table B** Characteristics of baseline HR-GNRs isolated from a rectal swab

| Patient | Micro-organism | Sequence Type | HR-GNR type^1^ | ESBL gene(s) | Subsequent HR-GNR infection (Y/N) |
| --- | --- | --- | --- | --- | --- |
| 1 | *K. pneumoniae* | 20 | ESBL | CTX-M-15 | N |
| 1 | *E. coli* | 1721 | Q&A | - | N |
| 2 | *E. coli* | 405 | ESBL | CTX-M-14 | Y |
| 2 | *E. coli* | 1642 | Q&A | - | Y |
| 3 | *E. cloacae complex* | 50 | ESBL | CTX-M-9 | N |
| 3 | *E. coli* | 88 | Q&A | - | N |
| 4 | *E. coli* | 6027 | ESBL | CTX-M-1, SHV-12 | N |
| 4 | *K. pneumoniae* | 16 | ESBL | CTX-M-1 | N |
| 5 | *E. coli* | 1798 | ESBL | CTX-M-14 | N |
| 5 | *K. pneumoniae* | 10 | ESBL | CTX-M-14 | N |
| 6 | *K. pneumoniae* | 15 | ESBL | CTX-M-15, SHV-28 | N |
| 6 | *E. coli* | 48 | ESBL | CTX-M-1 | N |
| 7 | *K. pneumoniae* | 22 | ESBL | CTX-M-15 | N |
| 7 | *E. coli* | 2178 | ESBL | CTX-M-3 | N |
| 8 | *E. cloacae complex* | 851 | ESBL | CTX-M-15, OXA-48 | N |
| 8 | *E. coli* | 162 | ESBL | CTX-M-15 | N |
| 9 | *M. morganii* | NA | Q&A | - | N |
| 10 | *E. coli* | ND | Q&A | - | N |
| 11 | *E. coli* | 10 | Q&A | - | N |
| 12 | *E. coli* | 1771 | Q&A | - | N |
| 13 | *E. coli* | 131 | Q&A | - | N |
| 14 | *E. coli* | 1721 | Q&A | - | N |
| 15 | *E. coli* | 10 | Q&A | - | N |
| 16 | *E. coli* | 648 | Q&A | - | N |
| 17 | *E. coli* | 354 | Q&A | - | N |
| 18 | *E. coli* | 1193 | Q&A | - | N |
| 19 | *E. coli* | 2599 | Q&A | - | N |
| 20 | *E. coli* | 57 | Q&A | - | N |
| 21 | *E. coli* | 131 | Q&A | - | N |
| 22 | *E. coli* | 648 | Q&A | - | N |
| 23 | *E. coli* | 69 | Q&A | - | N |
| 24 | *E. coli* | 131 | Q&A | - | Y |
| 25 | *E. coli* | 69 | Q&A | - | N |
| 26 | *K. pneumoniae* | 2459 | ESBL | CTX-M-15 | N |
| 27 | *K. pneumoniae* | 215 | ESBL | CTX-M-15 | N |
| 28 | *K. pneumoniae* | 1017 | ESBL | CTX-M-27 | Y |
| 29 | *K. pneumoniae* | 45 | ESBL | CTX-M-15, SHV-12 | N |
| 30 | *K. pneumoniae* | 45 | ESBL | CTX-M-15 | N |
| 31 | *K. pneumoniae* | 48 | ESBL | CTX-M-15 | N |
| 32 | *E. coli* | 6900 | ESBL | CTX-M-14 | N |
| 33 | *E. coli* | 635 | ESBL | SHV-12 | N |
| 34 | *E. coli* | 88 | ESBL | CTX-M-15 | N |
| 35 | *E. coli* | 540 | ESBL | CTX-M-15 | N |
| 36 | *E. coli* | 131 | ESBL | CTX-M-27 | N |
| 37 | *E. coli* | 10 | ESBL | SHV-12 | N |
| 38 | *E. coli* | 95 | ESBL | TEM-12 | N |
| 39 | *E. coli* | 410 | ESBL | CTX-M-15 | N |
| 40 | *E. coli* | 648 | ESBL | CTX-M-14 | N |
| 41 | *E. coli* | 10 | ESBL | CTX-M-15 | N |
| 42 | *E. coli* | 131 | ESBL | CTX-M-27 | Y |
| 43 | *E. coli* | 349 | ESBL | CTX-M-15 | N |
| 44 | *E. coli* | 131 | ESBL | CTX-M-27 | N |
| 45 | *E. coli* | 744 | ESBL | CTX-M-14 | N |
| 46 | *E. coli* | 131 | ESBL | CTX-M-15 | Y |
| 47 | *E. coli* | 5135 | ESBL | NA | N |
| 48 | *E. coli* | 227 | ESBL | CTX-M-15 | N |
| 49 | *E. coli* | 38 | ESBL | CTX-M-15 | N |
| 50 | *E. coli* | 86 | ESBL | CTX-M-1 | N |
| 51 | *E. coli* | 442 | ESBL | CTX-M-1 | N |
| 52 | *E. coli* | 349 | ESBL | CTX-M-1 | N |
| 53 | *E. coli* | 131 | ESBL | CTX-M-15 | N |
| 54 | *E. coli* | 131 | ESBL | CTX-M-9 | N |
| 55 | *E. coli* | 10 | ESBL | CTX-M-14 | N |
| 56 | *E. coli* | 635 | ESBL | SHV-12 | N |
| 57 | *E. coli* | 131 | ESBL | CTX-M-27 | N |
| 58 | *E. coli* | 69 | ESBL | CTX-M-1 | N |
| 59 | *E. coli* | 93 | ESBL | CTX-M14B | N |
| 60 | *E. coli* | 349 | ESBL | CTX-M14B | N |
| 61 | *E. coli* | 131 | ESBL | CTX-M-27 | N |
| 62 | *E. coli* | 5929 | ESBL | CTX-M-1 | Y |
| 63 | *E. coli* | 685 | ESBL | CTX-M-1 | N |
| 64 | *E. cloacae complex* | 850 | ESBL | CTX-M-15 | N |
| 65 | *E. cloacae complex* | 50 | ESBL | CTX-M-9 | Y |
| 66 | *E. cloacae complex* | 849 | ESBL | NA | N |
| 67 | *E. cloacae complex* | 50 | ESBL | CTX-M-1 | N |
| 68 | *E. cloacae complex* | 421 | ESBL | CTX-M-9 | Y |

ESBL: Extended Spectrum Beta Lactamase; Q&A: isolates resistant towards Fluoroquinolones and Aminoglycosides; ND: not determined; NA: not available

1: results based on phenotypic testing

**Table C** Characteristics of HR-GNR colonized patients

| **Patient characteristics** | **Total (n=68)** | **HR-GNR infected (n=8)** | **HR-GNR not infected (n=60)** |
| --- | --- | --- | --- |
| Number of HR-GNR colonized patients | 68 (100) | 8 (100) | 60 (100) |
| ESBL positive | 57 (83.8) | 8 (100) | 49 (81.6) |
| Q&A positive | 13 (19.1) | 1 (12.5) | 12 (20.0) |
| CPE positive | 1 (1.5) | 0 (0) | 1 (1.7) |
| Other positive | 0 (0) | 0 (0) | 0 (0) |
| Sex (male) | 36 (52.9) | 4 (50.0) | 32 (53.3) |
| ICU admission | 6 (8.8) | 1 (12.5) | 5 (8.3) |
| Used antibiotics 6 months before baseline | 21 (30.9) | 4 (50) | 17 (28.3) |
| Used antibiotics at baseline | 37 (54.4) | 5 (62.5) | 32 (53.3) |
| Admitted before baseline (up to 1 year) | 43 (63.2) | 6 (75.0) | 37 (61.7) |
| Earlier infection (up to two years before baseline) | 35 (51.5) | 7 (87.5) | 28 (46.7) |
| Mean age in years (SD) | 68.2 (16.3) | 70.1 (17.3) | 60.0 (16.3) |
| Median time from start admission to sampling in days (range) at baseline | 5.5 (0-48) | 5.0 (1-35) | 5.5 (0-48) |

Data are presented as numbers (%) unless indicated otherwise.

HR-GNR: Highly Resistant Gram-negative Rod; ESBL: extended spectrum beta lactamase; Q&A: Enterobacteriaceae resistant to fluoroquinolones and aminoglycosides; CPE: carbapenemase producing Enterobacteriaceae.

**Table D** Number and type of (HR-GNR) infection(s) between HR-GNR colonized and not colonized patients

|  | **Total** | **HR-GNR colonized** | **HR-GNR not colonized** |
| --- | --- | --- | --- |
| Total number of subsequent infection(s) | 239 (100) | 21 (100) | 218 (100) |
| UTI | 145 (60.6) | 15 (71.5) | 130 (59.6) |
| BSI | 47 (19.7) | 2 (9.5) | 45 (20.6) |
| Other | 47 (19.7) | 4 (19.0) | 43 (19.8) |
| Total number of subsequent (HR-GNR) infection(s) | 27 (100) | 9 (100) | 18 (100) |
| UTI | 14 (51.9) | 5 (55.6) | 9 (50.0) |
| Uncomplicated UTI | 6 (22.2) | 3 (33.3) | 3 (16.7) |
| Complicated UTI | 8 (29.6) | 2 (22.2) | 6 (33.3) |
| BSI | 5 (18.5) | 1 (11.1) | 4 (22.2) |
| Urosepsis | 3 (11.1) | 0 (0) | 3 (16.7) |
| Other BSI | 2 (7.4) | 1 (11.1) | 1 (5.6) |
| Other | 8 (29.6) | 3 (33.3) | 5 (27.8) |
| Wound infection | 6 (22.2) | 2 (22.2) | 4 (22.2) |
| Other infections | 2 (7.4) | 1 (11.1) | 1 (5.6) |

Data are presented as number (%) of infections for the total group of infections or HR-GNR infections as indicated

UTI: urinary tract infection; BSI: bloodstream infection; Other: other infection than UTI or BSI.
